# Sex differences in brain glucose metabolism and Alzheimer's disease risk and progression

**DOI:** 10.1002/alz.71491

**Published:** 2026-06-10

**Authors:** Marjan Ramezan, Aphrodite Demetriou, Sara Nicole Burke, Ivan Nalvarte, Andrew C. Shin

**Affiliations:** ^1^ Neurobiology of Nutrition Laboratory Department of Nutritional Sciences College of Health & Human Sciences Texas Tech University Lubbock Texas USA; ^2^ Department of Neurobiology Care Sciences and Society Division of Neurogeriatrics Karolinska Institute Solna Sweden; ^3^ Department of Neuroscience College of Science and BIO5 Institute University of Arizona Tucson Arizona USA; ^4^ Human Molecular Aging Center Texas Tech University Lubbock Texas USA; ^5^ Center of Excellence for Translational Neuroscience & Therapeutics Texas Tech University Health Sciences Center Lubbock Texas USA

**Keywords:** aging, bioenergetics, hypometabolism, mitochondrial functions, sex hormones

## Abstract

Sex differences are increasingly recognized as central to the biology of Alzheimer's disease (AD), yet the mechanisms through which they shape brain metabolism and disease vulnerability remain incompletely understood. Brain glucose hypometabolism is a core hallmark of AD and emerges decades before clinical decline, but accumulating evidence indicates that its causes, timing, and functional consequences differ between women and men. In this review, we synthesize findings from neuroimaging, molecular, and cellular studies to examine how sex‐dependent regulation of glucose transport, glycolysis, and mitochondrial function interacts with aging and AD pathology. We highlight reinforcing evidence for a steeper and more pathology‐linked decline in mitochondrial glucose metabolism in females, particularly in the context of menopause and apolipoprotein E (*APOE) ε4* genotype. We identify major knowledge gaps at the level of cell type, brain region, and disease stage, and outline priorities for sex‐informed, mechanistically anchored research to enable metabolic‐precision interventions for AD risk and progression.

## Highlights

- Brain glucose hypometabolism in Alzheimer's disease is sex specific.
- Females show lower brain mitochondrial glucose metabolism in Alzheimer's disease.
- The female glucose hypometabolism interacts stronger with amyloid and tau pathology.
- Menopause and greater susceptibility to apolipoprotein E (*APOE) ε4* play a role in such female bias.
- Sex‐informed metabolic pathways offer targets for precision Alzheimer's interventions.

## 1 INTRODUCTION

Alzheimer's disease (AD) is the most prevalent neurodegenerative cause of dementia and refers to a course of progressive loss of memory and cognition.^1^ One in three individuals aged 85 years and above have AD, while the lifetime risk at age 45 is nearly twice as high in women as that in men, and women constitute two‐thirds of all U.S. cases.^1^ This sex disparity can be due to the longer life expectancy of women than men; however, research shows that AD incidence is higher in women and emerging data suggest that the biological pathways leading to disease may differ between sexes.^1^, ^2^, ^3^

The pathological features of AD include the deposition of amyloid β‐peptide (Aβ) in the brain parenchyma and cerebral vessels, intracellular aggregation of hyperphosphorylated tau protein, sustained neuroinflammatory responses, and gradual synaptic and neuronal degeneration.^1^, ^4^ In addition to these pathological features, brain glucose hypometabolism is a well‐established hallmark of AD, detectable decades before clinical symptoms emerge.^5^, ^6^, ^7^, ^8^ Neuroimaging studies in AD consistently demonstrate reduced glucose metabolism in the posterior cingulate cortex, precuneus, parietotemporal regions, and bilateral temporal and parietal lobes, with later extension into the frontal cortex while typically sparing the precentral gyrus and occipital lobe.^9^ Further, reduced glucose utilization is more closely related to cognition than brain amyloid accumulation and often mediates tau–cognition relationships.^10^, ^11^

Across preclinical and prodromal stages, however, evidence indicates that the natural history of these metabolic and cognitive changes is not uniform between women and men. Women often demonstrate greater cognitive resilience, particularly in verbal and episodic memory, despite comparable levels of Aβ, tau, or atrophy, which may delay clinical detection when sex‐independent thresholds are applied.^12^, ^13^ This advantage appears to diminish and even reverse at more advanced pathological stages, where women exhibit steeper metabolic decline, greater neurodegeneration in memory‐related regions, and faster cognitive deterioration, especially among apolipoprotein E *(APOE) ε4* carriers.^12^, ^13^, ^14^, ^15^, ^16^ Growing evidence suggests that these sex differences may be closely tied to how the brain uses energy. In women, changes in glucose metabolism appear to interact more strongly with Aβ pathology, potentially helping explain why they experience a greater pathological burden and are more likely to develop AD.^17^, ^18^

Although brain glucose hypometabolism is a well‐established feature of AD, it is still unknown whether its causes, timing, and consequences differ between women and men, or what biological mechanisms account for these differences. The central question guiding this review is: *How do sex‐dependent differences in brain glucose metabolism interact with amyloid, tau, mitochondrial function, endocrine aging, and genetic risk to shape AD risk and progression?* In addressing this question, we synthesize findings across neuroimaging, molecular, and translational studies to clarify where true sex‐specific biology is supported and where critical gaps remain.

## 2 BRAIN GLUCOSE METABOLISM AND BIOENERGETIC CHANGES IN THE AD BRAIN

### 2.1 Normal brain glucose metabolism and the effect of aging

The brain is a metabolically expensive organ, with glucose as its main fuel and neurons and astrocytes having the highest glucose demands.^19^ Glucose supply is tightly regulated by the neurovascular unit that coordinates cerebral blood flow with metabolic demand. Through activity‐dependent signaling, the neurovascular unit ensures that regions of active neuronal firing receive increased delivery of glucose and oxygen to match their energy needs.^20^ Glucose uptake into the brain occurs via facilitated transporters: glucose transporter 1 (GLUT1) and glucose transporter 3 (GLUT3).^21^ GLUT1 facilitates the transport of glucose at the blood–brain barrier (BBB) level and exists in two isoforms distinguished by molecular weight and glycosylation status: the 55‐kD (more glycosylated) form is predominantly found in the endothelial cells of brain blood vessels allowing glucose transport from blood into the brain, while the 45‐kD form (less glycosylated) is primarily expressed in oligodendrocytes and the perivascular end feet of astrocytes, facilitating glucose uptake from the perivascular space into astrocytes and supports local glycolysis and metabolic coupling within the brain parenchyma.^22^, ^23^ GLUT3 is the major neuronal glucose transporter with a low Michaelis–Menten constant (Km), which ensures a steady delivery of glucose to neurons even when interstitial glucose levels are low.^22^ Once inside brain cells, glucose is rapidly phosphorylated by hexokinase to form glucose‐6‐phosphate (G6P), then gets metabolized to generate pyruvate, adenosine triphosphate (ATP), and nicotinamide adenine dinucleotide (NADH) through glycolysis.^19^, ^24^ Under aerobic conditions, pyruvate from glycolysis enters mitochondria, converts to acetyl‐CoA by pyruvate dehydrogenase complex, proceeds through the tricarboxylic acid (TCA) cycle and oxidative phosphorylation (OXPHOS), which eventually produces many more ATP molecules.^19^ The brain is also known to enhance glycolysis over OXPHOS under normoxic conditions, which happens during neuronal activation, a phenomenon termed “aerobic glycolysis.^25^” Beyond energy production, glucose metabolism also supports biosynthesis. Some G6P is diverted into the pentose phosphate pathway (PPP), generating nicotinamide adenine dinucleotide phosphate (NADPH) and ribose sugars for antioxidant defense, and nucleic acid and lipid (myelin) synthesis.^26^

Neurons mainly rely on oxidative metabolism, sending most of their glucose through the TCA cycle to supply the energy needed to maintain ion gradients, fire action potentials, and synthesize and package neurotransmitters.^19^, ^27^ Astrocytes, on the other hand, characteristically have a higher rate of glycolysis relative to oxidation, partly to support their role in neurotransmitter clearance and to maintain their unique glycogen stores that serve as an important local energy buffer for neurons.^19^ Since the glycolytic rate in astrocytes is high, they can produce lactate and release it to be used by neurons especially during periods of high neuronal activity, a concept that has been proposed as the astrocyte‐neuron lactate shuttle.^28^ Lactate import into neurons is shown to be essential for forming long‐term potentiation (LTP) and long‐term memory.^29^ Indeed, glucose and lactate are exchanged between astrocytes and neurons in an activity‐dependent manner, and both molecules play critical roles as energy substrates for neurons. This tight metabolic coupling between astrocytes and neurons ensures that active synapses have an adequate and continuous fuel supply.^29^ Oligodendrocytes, the cells responsible for producing myelin, shunt a large amount of glucose into the PPP instead of glycolysis. This is because oligodendrocytes require NADPH and ribose‐5‐phosphate to synthesize fatty acids and cholesterol for myelin formation.^30^ Microglia, the brain's resident immune cells, also consume glucose and can upregulate glycolysis when activated. This shift reflects metabolic reprogramming; whereby proinflammatory microglia rely more heavily on glycolysis and reparative phenotypes favor OXPHOS. Thus, glucose metabolism plays a central role in regulating microglial function and phenotypic plasticity.^31^ Glucose metabolism in the brain is also heterogeneous across regions based on differences in cytoarchitecture, cell‐type composition, and functional activity. Generally, gray matter regions such as cortex, basal ganglia, and hippocampus that are rich in neuronal cell bodies and synapses are more metabolically active than white matter tracts like myelinated bundles of axons.^32^, ^33^, ^34^ The regional cerebral glucose metabolic rate is coupled with local neuronal activity and synaptic density at rest. Fluorodeoxyglucose positron emission tomography (FDG‐PET) is an imaging technique that measures regional brain glucose uptake (used as a proxy for glucose metabolism) using the radiolabeled glucose analog ^1^ ^8^F‐FDG. Studies using this technique show that the neocortex, association cortices, and basal ganglia have higher baseline glucose uptake, while other regions such as the cerebellum (under resting conditions) and white matter have lower rates.^35^, ^36^ Additionally, the hippocampus and cingulate cortex show high rates, reflecting their active roles in memory processing and integrative processes even at rest.^37^ Given the strong link between neuronal activity and glucose metabolism, healthy individuals show a decline in mental functions following glucose deprivation, manifested as difficulty concentrating, delayed reaction times, and memory lapses,^38^ which improve following glucose administration.^38^, ^39^, ^40^, ^41^, ^42^, ^43^, ^44^ Consistent with these findings, blocking glycogen breakdown in astrocytes or blocking lactate release to neurons in rats has been shown to impair long‐term memory formation.^29^ Apart from the glucose utilization rate itself, the metabolic fate of glucose differs by region. For example, the thalamus has lower glycolytic intermediates and higher PPP activity that help support NADPH production and lipid biosynthetic processes compared to the cortex and amygdala,^32^ perhaps due to its high oligodendrocyte‐rich cellular makeup and glutathione recycling by NADPH for redox protection.

After early adulthood, the brain's glucose metabolism gradually decreases with age even in the absence of any neurodegenerative diseases. Cerebral metabolic rate is lower in healthy older adults compared to that in younger adults, both globally and in specific brain regions.^45^, ^46^, ^47^, ^48^ Normal aging is associated with the largest reduction in glucose utilization in the frontal cortex, the striatum (caudate nucleus), entorhinal cortex, and hippocampus.^45^, ^46^ These patterns align with the notion that frontal cortices, involved in executive function and working memory, and the temporal lobe involved in auditory processing, language comprehension, and memory are particularly vulnerable to the aging process (“frontal aging hypothesis”^49^) in terms of metabolic activity. Importantly, even after accounting for brain atrophy occurring in older adults, the metabolic efficiency per gram of tissue remains lower in the key cortical areas, indicating true functional hypometabolism and not just fewer numbers of neurons.^46^ In addition to changes in metabolic activity, alterations in glucose transporter expression may contribute to age‐related reductions in cerebral glucose utilization.^50^, ^51^ Experimental studies in rodents have reported declines in GLUT1 expression at the BBB^50^, ^51^, ^52^ and reductions in neuronal glucose transporters, including GLUT3 and GLUT4,^50^, ^53^ with advancing age, accompanied by decreased brain glucose uptake. However, these changes appear to be modest and regionally variable, with some studies reporting only slight or non‐significant reductions in GLUT3 or no consistent changes in GLUT1 and GLUT3 across all brain regions or models.^54^, ^55^ This is in contrast with more pronounced and region‐specific reductions in GLUT1 have been observed in AD models, particularly in the hippocampus, independent of vascular density changes and associated with amyloid pathology.^51^, ^55^ On the contrary, certain regions like the cerebellum and brainstem tend to show more consistent, or even slightly increased, relative glucose uptake as a function of chronological aging.^48^ The brain's ability to meet cognitive demands may be diminished by the age‐related decline in glucose utilization, especially in metabolically active areas. This could also make the brain more susceptible to neurodegenerative processes like AD.^56^, ^57^

### 2.2 Glucose metabolism dysregulation in AD

Brain glucose hypometabolism is an invariant feature of AD,^8^, ^58^, ^59^, ^60^, ^61^, ^62^, ^63^, ^64^ which exists decades before cognitive decline^65^, ^66^, ^67^ and brain atrophy.^68^ This observation has been consistently reported in both human and animal models of AD.^8^, ^58^ Brain glucose hypometabolism may not only precede AD pathology but also actively contribute to its development.^56^ The decline in cerebral metabolic rate of glucose, mainly measured with FDG‐PET, is mostly seen in parietal and temporal lobes,^60^, ^61^, ^62^, ^64^, ^65^, ^66^ as well as limbic system,^63^ precuneus, and cingulate cortex^59^, ^64^ of AD patients. However, it is suggested to involve memory‐related brain areas, such as the entorhinal cortex and hippocampus, before proceeding to other areas.^7^ FDG‐PET mainly reflects the net glucose uptake into brain tissue and is governed by glucose transport and intracellular phosphorylation by hexokinase, which traps FDG as FDG‐6‐phosphate.^69^ Although widely interpreted as a proxy for neuronal metabolic activity, the FDG signal reflects a composite of cellular and metabolic processes.^25^, ^69^ Since FDG uptake captures early glycolytic steps rather than downstream mitochondrial oxidation, it is strongly influenced by cell types with high glycolytic activity. Autoradiographic and tracer studies demonstrate that astrocytes, which exhibit high glycolytic rates and are tightly coupled to synaptic activity, contribute substantially to FDG uptake and may dominate the signal under certain conditions.^70^ In addition, activated microglia and neuroinflammatory processes, which involve a shift toward glycolytic metabolism, can further modulate FDG signal, particularly in neurodegenerative conditions such as AD.^71^

Among various GLUTs, GLUT1 and GLUT3 have been implicated in the development of AD.^72^, ^73^, ^74^, ^75^ GLUT1 and GLUT3 are consistently decreased in cortical and hippocampal regions in AD and in transgenic amyloid models.^75^ High local Aβ levels associate negatively with these transporters and are likely a driver of their reductions.^75^, ^76^, ^77^, ^78^ Additionally, a reduction in these two glucose transporters is linked with decreased O‐GlcNAcylation that leads to tau becoming excessively phosphorylated, contributing to the development of neurofibrillary tangles and neurodegeneration.^72^ GLUT3 reduction, in particular, is shown to precede the onset of clinical symptoms.^74^ Increased cellular calcium in AD is implicated to reduce GLUT3 gene transcription in human prefrontal cortex through a cAMP response element‐binding protein (CREB) mechanism.^76^ Recent findings suggest that Aβ peptides may disrupt GLUT1 at BBB and contribute to cerebral hypometabolism in AD. In transgenic APP/PS1 mice, Aβ peptides promote GLUT1 internalization into the cytoplasm and degradation by augmenting insulin‐AKT‐TXNIP pathway, leading to reduced glucose transcytosis across the BBB.^77^ Downregulation of the main brain GLUTs—GLUT1 and GLUT3—in the AD brain is accompanied by cell‐type‐specific changes in alternative transporters, including increased expression of GLUT2 in astrocytes, GLUT4 in neurons, and GLUT12 in glial populations such as astrocytes and oligodendrocyte‐lineage cells. These changes likely represent a compensatory response; however, they are insufficient to restore glucose homeostasis, as GLUT1 and GLUT3 remain the principal transporters mediating glucose entry into the brain and neuronal uptake. In addition, these alternative transporters differ in their regulation and subcellular localization which further limits their ability to fully compensate for impaired glucose transport. For instance, GLUT4 is insulin dependent, and GLUT12 is largely intracellular and translocates in response to signals such as glucose, insulin, and inflammatory cytokines. While some associations with gliosis or AD pathology have been reported, the functional consequences of their upregulation remain unclear and are not definitively established as either beneficial or detrimental.^54^, ^75^, ^79^

Aerobic glycolysis is postulated to play a protective or adaptive role in response to early AD pathology, perhaps helping the brain stay functional despite some Aβ buildup. Recent evidence highlights the importance of aerobic glycolysis in the aging brain and its potential relationship to early disease processes.^80^, ^81^, ^82^ In young healthy individuals, aerobic glycolysis is high in the medial prefrontal cortex, posterior cingulate cortex, lateral parietal cortex, and lateral temporal cortex which process cognitive functions.^80^ In this context, aerobic glycolysis is not measured directly but is derived from PET‐based estimates of glucose uptake (measured by FDG‐PET) and oxygen consumption (measured by ^15^O‐O_2_ PET), representing the fraction of glucose metabolism that exceeds OXPHOS. Thus, while FDG‐PET reflects total glucose uptake, aerobic glycolysis specifically captures non‐oxidative glycolytic flux, allowing these processes to be dissociated. Using this framework, aerobic glycolysis is low in age‐related cognitive impairment, especially in the presence of Aβ. In contrast, Aβ presence without cognitive decline is associated with elevated aerobic glycolysis, but when the white matter hyperintensity is high, aerobic glycolysis declines even in cognitively normal individuals. These findings indicate that early, asymptomatic Aβ deposition is associated with a likely compensatory increase in brain aerobic glycolysis whereas vascular burden and cognitive impairment are concomitant with dampened aerobic glycolysis.^80^ Heightened glycolysis has also been shown in AD neurons and astrocytes, and neurons take on a pro‐apoptotic feature as a result of Warburg‐like metabolic transformation.^81^, ^83^ In AD, a shift in pyruvate kinase isoform expression has been observed, with greater presence of the pyruvate kinase M2 isoform in hippocampal tissue and human induced pluripotent stem cell (iPSC) ‐derived neurons from AD patients. This isoform is associated with heightened glycolysis and neuronal fate loss.^81^ Such a Warburg‐like shift from OXPHOS to aerobic glycolysis has also been shown in response to human tau in *Drosophila* neurons. Knocking down glycolytic and lactate dehydrogenase genes in this model lowered tau‐induced premature lethality and improved aging rate.^84^ Hence, the adaptive role of heightened glycolysis does not seem to be a persistent metabolic resolution; rather, it can drive AD pathology leading to severe cognitive decline.

The glycolysis pathway can also become impaired in AD,^74^, ^85^, ^86^ and Aβ and tau pathology can initiate such impairment.^85^, ^86^ Infusion of soluble Aβ1‐42 oligomers into the mouse brain activates NADPH oxidase 2 (NOX2) and leads to generation of reactive oxygen species (ROS) that in turn suppresses glycolytic flux. NOX2 activation is accompanied by neuronal hyperexcitability and higher aggression and anxiety in these mice.^86^ Amyloid and tau oligomers have also been implicated to suppress hippocampal glycolysis by inducing the enzyme indoleamine‐2,3‐dioxygenase 1 (IDO1) which converts tryptophan to kynurenine.^85^ Kynurenine over‐production suppresses hypoxia‐inducible factor‐1 alpha (HIF‐1α) nuclear signaling necessary for glycolysis in astrocytes.^85^ The ensuing astrocytic glycolytic deficit can reduce lactate production, which can otherwise be delivered to neurons for oxidative metabolism and synapse function, leading to impaired neuronal LTP and cognition. While not influencing total cerebral Aβ40 and Aβ42 load in mice, IDO1 inhibition reverses glucose metabolic abnormalities in mouse primary and human iPSC‐derived astrocytes and neurons, improves cognition, and decreases aggregated plaques in the subiculum and cortex of AD mice.^85^ Diminished glycolytic flux along with higher brain tissue glucose concentration and lower GLUT3 levels are also shown to be associated with more severe AD pathology and the emergence of AD symptoms in the *post mortem* cortical tissue from a cohort of AD participants. It may suggest that reductions in glucose utilization by cells may contribute to glucose accumulation in the brain, promote the progression of AD pathology, and ultimately lead to the onset of clinical symptoms.^74^ In addition, individuals with high amyloid burden who have lower aerobic glycolysis experience accelerated tauopathy in a multi‐tracer PET study.^82^ Hence, although glycolysis is enhanced in response to AD pathology in the early stage as an adaptive mechanism, more severe Aβ buildup can dampen glycolysis through different mechanisms. Once glycolytic activity declines, lactate production and LTP may also weaken, and together with other abnormalities, these changes can ultimately contribute to cognitive decline.

A growing body of evidence suggests that mitochondrial dysfunction is a central contributor to the impaired glucose metabolism observed in AD.^87^, ^88^ In AD, the enzymatic activity of the Krebs cycle, respiratory chain function, and ATP production is reduced while oxidative stress increases.^87^ Post‐mortem analyses of AD brains reveal decreased cytochrome oxidase (Complex IV) activity in the frontal, temporal, parietal, and occipital cortices.^89^ In line with this, findings from 11‐month‐old male 3xTg‐AD mice show mitochondrial complex downregulation and glucose hypometabolism prior to overt amyloid and tau pathology in piriform and insular cortices, highlighting bioenergetic dysfunction as an early feature that may initiate a cascade of metabolic vulnerability prior to neuropathological features.^90^ Defects in OXPHOS—notably Complex I subunits—are evident in transcriptomic^91^, ^92^ and proteomic^93^, ^94^ analyses of both neurons and glia from human AD brains. Mitochondrial proteome alterations are especially prominent in late‐stage AD, and this may actually reflect a progressive metabolic deterioration since the changes are also detectable early in mild cognitive impairment (MCI) stage.^93^ While the causal direction remains unresolved, the presence of these mitochondrial abnormalities early in the disease trajectory supports the hypothesis that energy metabolism defects are not merely consequences of Aβ and tau, but may instead sensitize the brain to these pathological features. Age‐related declines in mitochondrial efficiency, together with the gradual buildup of metabolic disturbance (biological entropy), undermine the brain's ability to maintain stable energy balance in AD.^95^ The brain, as a highly energy‐demanding organ, relies on mitochondria to sustain low‐entropy ordered neural activity. As the mitochondrial capacity declines with age, internal entropy increases, leading to reduced neuronal adaptability, collapse of cognitive reserve, and eventual neurodegeneration.^95^ Demetrius and Driver^96^ formalized this concept through the entropic selection hypothesis, which frames aging and sporadic AD as outcomes of evolutionary and thermodynamic forces acting on energy metabolism. Their model posits that, under conditions of limited but constant energy availability, neurons experiencing mitochondrial impairment undergo metabolic reprogramming by upregulating OXPHOS, termed as “inverse Warburg effect,” as a survival strategy. This hypermetabolic state gives a temporary competitive advantage and allows these neurons to outcompete normal cells for energy substrates. However, this adaptation imposes long‐term costs by increasing oxidative stress and functional decline, ultimately pushing the system toward pathological aging.^97^, ^98^ In AD transgenic mice (e.g., Tg2579 and PS1/A246E) increased expression of genes involved in OXPHOS such as subunits of mitochondrial complexes I, III, IV, and V was observed even before the onset of Aβ pathology and cognitive decline.^97^, ^98^ An increase in brain glucose uptake is also observed in widely used AD mouse models (Tg2579, APP/PS1, 5xFAD, 3xTg) during the early stages of pathology.^99^, ^100^, ^101^, ^102^ Interestingly, several studies have detected brain hypermetabolism at very early stages in some brain regions in MCI patients.^103^, ^104^, ^105^, ^106^, ^107^, ^108^, ^109^ Similarly, some studies of human AD brains have revealed elevated expression of mitochondrial complex IV subunits, along with increased mitochondrial DNA levels in the hippocampus and cortex of individuals with sporadic AD.^110^, ^111^, ^112^, ^113^ Despite these early enhancements, mitochondrial function declines when Aβ and tau pathology become pervasive.^114^ Aβ and tau pathology can also cause mitochondrial dysfunction. Aβ can promote cyclophilin D‐dependent mitochondrial permeability transition pore formation, which triggers loss of membrane potential, Ca^2^^+^ dysregulation, ROS, impaired respiration,^115^ and hamper with axonal mitochondrial trafficking.^114^ Disease‐associated/phosphorylated tau can also disrupt mitochondrial transport, dynamics, and bioenergetics, contributing to neuronal vulnerability.^116^ In a feed‐forward manner, mitochondrial dysfunction can lead to more Aβ deposition and more tau phosphorylation.^117^ Mitochondrial targeted antioxidants, such as MitoQ (a ROS quencher) or EUK189 (a superoxide dismutase mimetic), have been able to reduce Aβ accumulation alongside functional benefits in AD mice,^117^, ^118^ reinforcing the role of oxidative stress in AD pathology aggregation. Furthermore, oxidative stress resulting from mitochondrial bioenergetic dysfunction is linked to increased BACE1 (β‐secretase) expression and activity and amyloidogenic APP processing, offering a plausible mechanistic route by which mitochondrial ROS can amplify Aβ generation.^119^

Brain insulin resistance is a feature of AD and has emerged as a mechanism that promotes glucose metabolism dysregulation in AD. Insulin supports neuronal survival, synaptic plasticity, and energy metabolism via insulin receptor‐mediated signaling pathways such as PI3K‐Akt and MAPK.^120^ However, these signaling pathways are disrupted in AD in a way that can promote tau hyperphosphorylation and neurotoxicity.^120^, ^121^ In post‐mortem AD brain tissue, reduced insulin receptor density, altered isoform distribution, and impaired downstream signaling are consistently evidenced even in individuals without diabetes, suggesting that the brain insulin resistance is intrinsic to AD pathology.^121^, ^122^ In particular, GLUT4, which is recruited to the neuronal membrane during high metabolic demand (e.g., learning), becomes less responsive to insulin, thereby reducing glucose flux in neurons when it is most needed.^120^ Downregulated insulin signaling in AD also occurs in astrocytes where it is needed to regulate glycogen storage and lactate export, further compounding the energy shortfall in neurons that are dependent on astrocytic lactate shuttling.^120^, ^123^ These impairments are especially prominent in metabolically active and memory‐related regions such as the hippocampus and cortex, which overlap spatially with the areas of hypometabolism detected by FDG‐PET imaging in early AD.^124^, ^125^, ^126^, ^127^ Insulin resistance is shown to reduce brain glucose uptake as measured by FDG‐PET in individuals with impaired glucose tolerance.^128^ Impaired insulin transport across the BBB, lowered expression of insulin receptor, and enhanced insulin‐degrading enzyme activity exacerbate central insulin insufficiency, leading to reduced cerebral glucose utilization, as well as decreased Aβ clearance and worsened tau pathology.^120^, ^121^

Importantly, the relationship between glucose hypometabolism and AD pathology is likely bidirectional rather than strictly linear.^64^, ^65^, ^68^, ^75^, ^77^, ^78^, ^129^ While the evidence above demonstrates that Aβ can impair glucose metabolism through mechanisms such as reduced GLUT expression and impaired glycolytic function, metabolic dysfunction such as mitochondrial OXPHOS impairment may also precede and contribute to the development of AD pathology. Thus, rather than representing a purely downstream consequence of amyloid and tau pathology, impaired glucose metabolism may act both as an early vulnerability factor and as a process further exacerbated by disease progression.

**FIGURE 1:**
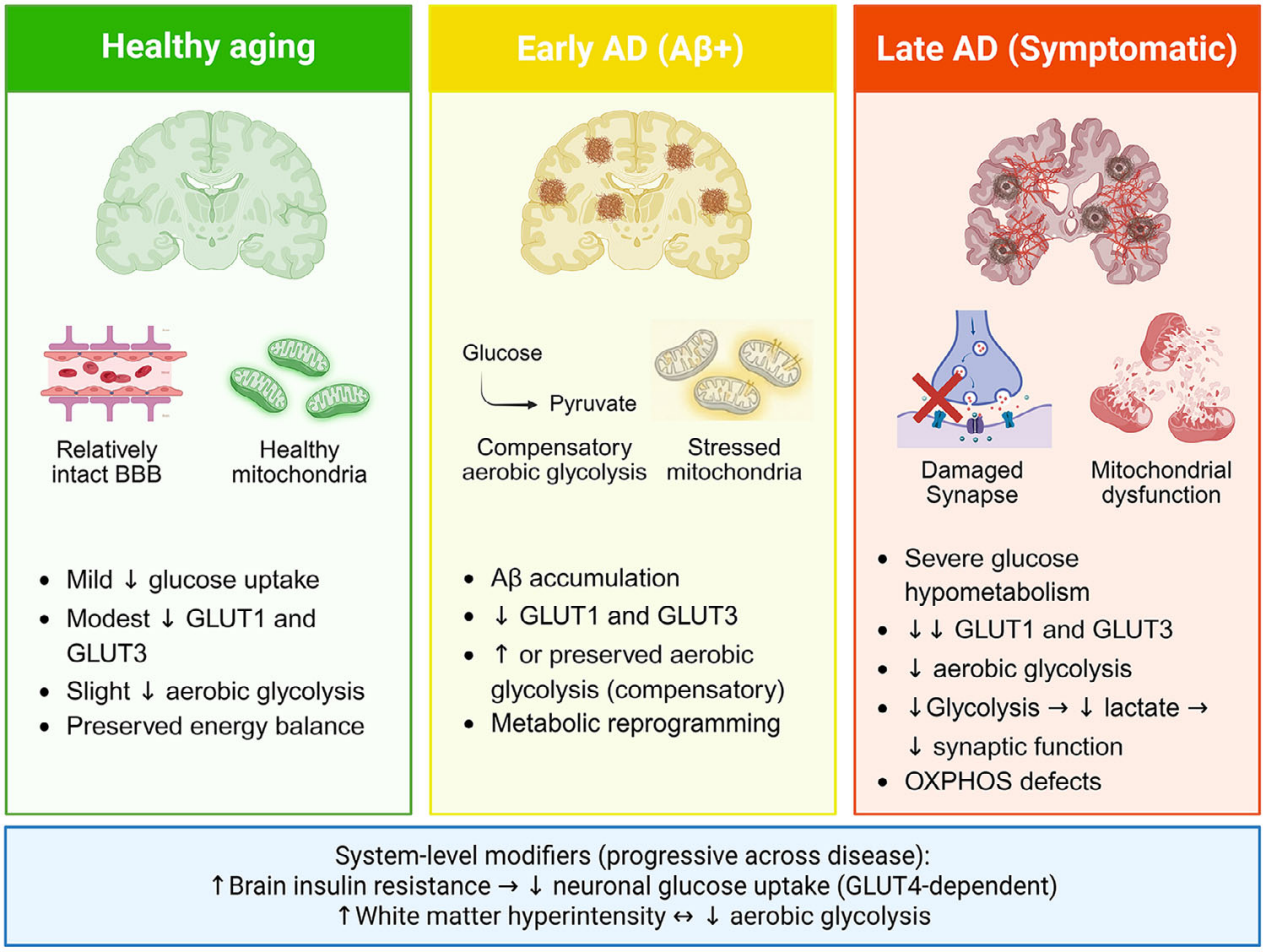
Temporal progression of glucose metabolic alterations across healthy aging and AD. ↑, increase; ↓, reduction; →, leads to; AD, Alzheimer's disease.

Figure 1 illustrates the progression of glucose metabolic changes across healthy aging and AD.

## 3 SEX DIFFERENCES IN BRAIN GLUCOSE METABOLISM IN HEALTHY AGING AND AD

### 3.1 Sex‐specific glucose metabolic alterations in healthy aging

Across cognitively normal and early‐stage AD, females display a persistently younger metabolic profile than males, characterized by higher glucose utilization and aerobic glycolysis.^130^, ^131^, ^132^, ^133^ This pattern reflects a greater maintenance of youthful glycolytic metabolism in females, suggesting slower metabolic aging and potentially better resilience to age‐related decline. The sex difference is primarily driven by glucose‐related parameters rather than cerebral blood flow or oxygen consumption, and appears independent of amyloid burden.^130^ Sex affects regional brain glucose metabolism in a wide age range as shown in cross‐sectional studies.^134^, ^135^, ^136^ Men are shown to have higher metabolism in brain regions such as frontal, temporal, anterior parietal, and insular cortices as well as cerebellum. Females, on the other hand, exhibit higher metabolism mainly in the posterior regions such as posterior parietal and occipital lobes, thalamus, hypothalamus, and hippocampus, as well as some minor anterior parts.^134^, ^135^, ^136^ Of note, some of these studies are limited by small sample size, including study participants from a wide age range, and used uncorrected *p*‐values for voxel‐wise threshold. These methodological choices create a false positive risk. Furthermore, no partial volume correction was implemented, so the reported sex differences could be attributed to changes in both metabolism and atrophy.^134^, ^136^

In addition to these cross‐sectional studies, longitudinal studies have been conducted to capture age‐related glucose‐metabolic decline in both sexes.^137^, ^138^ Voxel‐wise FDG‐PET analysis of the whole brain showed a glucose metabolism decline after age 60 in frontal cortex, anterior cingulate, and temporal cortex in both sexes.^137^ In addition, males and females had a more severe decline by aging in caudate nucleus and occipital lobe region, respectively. Additionally, the metabolic decline after age 70 was worse in males than females.^137^ Such male‐skewed glucose metabolic decline by aging is also shown by Malpetti et al.,^138^ especially in the medial frontal cortex and anterior cingulate gyrus. Females, however, experience a less extensive age‐related hypometabolism during normal aging.

### 3.2 Sex‐specific brain glucose metabolism in AD

Sex differences in brain glucose metabolism are increasingly recognized as critical factors in aging and AD pathogenesis. Table 1 summarizes the studies that have addressed this issue in AD.

**TABLE 1:** Studies on sex differences in brain glucose metabolism in Alzheimer's disease

Reference	Model	Region or cell type of sex difference	Technique	Sex‐specific effect	Affected pathway, complex or enzyme
Bouter et al.^130^	5XFAD transgenic mice (7 months; ♂♀)	Cortex, hippocampus, thalamus, cerebellum, midbrain	^1^ ^8^F‐FDG PET/MRI	↓ glucose metabolism in ♀ vs. ♂ and WT	Glucose metabolism (FDG uptake)
Gherardelli et al.^133^	APP/PS1 transgenic mice (4, 8, 11 months; ♂♀)	Hippocampus	Radiolabeled glucose assays (D‐[3‐^3^H]‐glucose for glycolytic flux)	♀ ↓ glycolytic flux at 8 & 11 months vs. 4 months AD; ♂ = no change in AD	Glycolysis
Guillot‐Sestier et al.^134^	APP/PS1 transgenic mice (9 months; ♂♀)	Microglia from whole brain tissue	Transcriptomics, metabolomics, Seahorse for glycolytic flux	♀ ↑ glycolysis, ↑ glycolytic enzyme expression, ↑ lactate in AD; ♂ = no change in AD	Glycolysis, TCA cycle (succinate accumulation)
Flannagan et al.^75^	Human iPSC‐derived neurons, astrocytes, and organoids (♂♀, sporadic AD vs. control)	Neurons and astrocytes (forebrain‐like)	Seahorse metabolic flux analysis, COX Vmax assay, mitochondrial imaging (TMRE, MitoSOX), Western blot	♀: ↓ Complex IV activity (neurons/organoids), ↑ mitochondrial Ca^2^^+^ & ROS, ↑ mitochondrial membrane potential, ↑ mitochondria–lysosome colocalization (astrocytes) ♂: Mitochondrial depolarization, ↓ mitochondrial mass (neurons) ♂♀: OXPHOS → glycolysis shift	OXPHOS Glycolysis Mitophagy
Park et al.^17^	Human subjects with AD (Cohort 1: *n* = 181, FDG‐PET, PiB‐PET, MRI, plasma biomarkers); postmortem brains (Cohorts 2–3: *n* = 96)	Whole brain (imaging); middle temporal gyrus & prefrontal cortex (RNA‐seq)	FDG‐PET, MRI, plasma Aβ42/40 ratio, transcriptomics	♀ ↓ glucose metabolism; ♀ > > ♂ in DEGs (mostly down‐regulation); ♀ glycolysis, carbon metabolism, and AMPK signaling genes are significantly altered in AD	Glucose metabolism (FDG uptake), Glycolysis/gluconeogenesis, AMPK, insulin signaling pathways linked to APP & MAPT dysregulation
Sundermann et al.^124^	Human subjects (*n* = 1259; 55–90 years; ADNI dataset)	FDG‐PET ROIs: bilateral posterior cingulate, bilateral angular gyri, middle/inferior temporal gyrus	FDG‐PET SUVR, MRI hippocampal volume, Aβ‐PET (AV45), cognitive tests (MMSE, RAVLT, TMT‐B)	Glucose metabolism was ♀ > ♂ at mild to moderate pathology and the advantage absent at severe pathology; ♀ higher metabolism → better cognition (MMSE, memory, exec. function); steeper metabolic decline later	Glucose metabolism (FDG uptake)
Nerattini et al.^138^	Human AD patients (*n* = 247; 151♀, 96♂)	Whole‐brain; voxel‐wise analysis	FDG‐PET (SPM12 voxel‐based analysis, FWE‐corrected), MRI co‐registration	Regional glucose hypometabolism (frontal/limbic in ♀; parietal in ♂)	Glucose metabolism (FDG uptake)
Demarest et al.^131^	3×Tg‐AD mice, 18 months (♂♀)	Cortex (synaptic & non‐synaptic mitochondria)	Seahorse respirometry (complex I–IV), Western blot (ETC subunits)	♀ ↓ Complex I (NDUFB8, MTCO1, UQCRC2), ↑ non‐synaptic Complex II (SDHB); ♂ ↑ Complex IV, ↑ UQCRC2 & SDHB	OXPHOS
Adlimoghaddam et al.^132^	3×Tg‐AD and WT mice (2, 6, 13 months; ♂♀)	Hypothalamus (isolated mitochondria)	Seahorse OCR, Western blot (OXPHOS I–V), COX activity assay, cytokine multiplex, WB for NF‐κB, pIκB‐α, Nrf2	OCR: ♀ ↓ (from 2 months) vs. ♂ ↓ (from 6 months); Complex II/III subunits: ♀ ↓ vs. ♂ no change; COX activity: ♀ ↓ vs. ♂ no change; NF‐κB/pIκB‐α/Nrf2: ♀ ↑ vs. ♂ no change	OXPHOS NF‐κB/Nrf2
Soudy et al.^139^	Human *post mortem* AD and control brains (♂♀)	Prefrontal cortex	Single‐nucleous RNA‐seq	**♀**: ↑ expression of mitochondrially encoded complex I genes (MT‐ND1–ND6) in excitatory neurons; **♂**: ↓ complex I genes in excitatory neurons; ↓ MT‐CO1 (complex IV) in astrocytes	OXPHOS
Yang et al.^140^	Human postmortem AD & control brains (♂♀)	Prefrontal cortex (Brodmann area 10)	Western blot, Seahorse respirometry, cytokine assay, Immunohistochemistry	♀: ↓ Complex III & IV respiration (greater decline in OCR); ♂: ↑ p‐mTOR S2448 (insulin signaling), no significant OCR decline	OXPHOS Akt/mTOR signaling Inflammatory activation
Gallart‐Palau et al.^141^	Human *post mortem* AD + CVD and control brains (♂♀)	Temporal cortex (BA21 white matter and mitochondria proteome)	Quantitative proteomics, Western blot	♀: ↑ deamidation & citrullination of MBP → impaired MBP degradation and accumulation; ↓ glutamine synthetase, ↓ ATP synthase, ↓ cytochromes, ↓ NADH‐ubiquinone α‐subunits; ↑ NADPH (promotes deamidation) ♂: No significant MBP deamidation/citrullination; relative preservation of mitochondrial proteins	MBP hyper‐deamidation / citrullination OXPHOS Glutamine‐glutamate metabolism
Perneczky et al.^123^	Human patients with AD (*n* = 93; 43♀, 50♂)	Inferior frontal and superior temporal gyri, the hippocampus, and the insular cortex in the right hemisphere	^18^F‐FDG PET imaging	Glucose metabolism was ♀ > ♂ when cognitive function was controlled for	Glucose metabolism (FDG uptake)
Maffioli et al.^137^	Human postmortem AD and control brains (♂♀)	Hippocampus	Metabolomics Proteomics	Healthy state ♀ > ♂: HIF‐1α, TCA cycle, and Erβ signaling AD state ♀ < ♂: HIF‐1α, TCA cycle, and Erβ signaling, Response to insulin stimulus, GLP‐1 regulation of insulin secretion	HIF‐1α, TCA cycle, and Erβ signaling pathways Response to insulin stimulus, GLP‐1 regulation of insulin secretion

Abbreviations: ♀, females; ♂, males; ↑, increase; ↓, reduction; →, leads to; AD, Alzheimer's disease; ADNI, Alzheimer's Disease Neuroimaging Initiative; COX, cyclooxygenase; DEG, differentially expressed gene; FDG‐PET, fluorodeoxyglucose positron emission tomography; GLP‐1, glucagon‐like‐peptide‐1; HIF‐1α, hypoxia‐inducible factor‐1; MBP, myelin basic protein; MMSE, Mini‐Mental State Examination; MRI, magnetic resonance imaging; NADH, nicotinamide adenine dinucleotide; NADPH, nicotinamide adenine dinucleotide phosphate; OCR, oxygen consumption rate; OXPHOS, oxidative phosphorylation; ROI, region of interest; SUVR, standardized uptake value ratio; TCA, tricarboxylic acid; TMT, Trail Making Test.

Preclinical studies show sex differences in brain glucose metabolism in AD across different brain regions.^139^, ^140^, ^141^, ^142^ Seven‐month‐old female 5xFAD mice have shown lower glucose metabolism in the cortex, hippocampus, thalamus, cerebellum, and midbrain than their male counterparts as measured by FDG‐PET/magnetic resonance imaging (MRI) imaging.^139^ All of these brain regions except cerebellum had already developed amyloid pathology equally in both sexes,^139^ implying that amyloid pathology might affect glucose metabolism in a sex‐dependent manner. Sex differences in hippocampal glucose metabolism are also observed in APP/PS1 mouse model,^142^ where glycolytic flux measured by the rate of ^3^H_2_O production from D‐[3‐^3^H]‐glucose is reduced at 8‐ and 11‐months of age compared to 4‐months in female mice, but not in male APP/PS1 mice. Radioactive tracing of D‐[1‐^14^C] glucose used as a measure for brain glucose entry, however, reveals no sex or age differences.^142^ Besides being sex‐dimorphic, glycolysis regulation in AD can be cell‐type dependent. Microglial metabolism in 18‐month‐old APP/PS1 mouse model shows female‐specific upregulation of glycolysis, increased expression of glycolytic enzymes, and elevated lactate production, while TCA cycle flux alterations (e.g., succinate accumulation) further suggest a sex‐dependent shift in the central carbon metabolism that favor glycolysis over OXPHOS in female microglia.^143^ The glycolytic microglia cannot perform their normal phagocytic and chemotactic functions, which in turn reduces their ability for amyloid clearance and increases brain inflammation.^143^ Supporting these findings, microgliosis is shown to be more severe in old female than male APP/PS1 mouse hypothalamus. Such a dysfunction is accompanied by exacerbated Aβ burden and a high GLUT1 expression.^144^ Hypothalamus is suggested as a primary region of metabolic abnormalities in AD.^145^ Interestingly, hypothalamic GLUT1 gene expression decreases in young and increases in old female AD mice compared with their age‐matched male counterparts, indicating a possible sex‐dimorphic compensatory shift in the hypothalamic glial glucose metabolism.^144^

In a study comparing 93 male and female individuals with mild AD, the inferior frontal and superior temporal gyri, hippocampus, and insular cortex in the right hemisphere show lower FDG‐PET uptake values in men than women when cognitive function—measured by the Consortium to Establish a Registry for AD neuropsychological battery—was controlled for.^132^ This suggests that compared to women, men may be better at compensating for the metabolic deficit in AD, and their brain glucose hypometabolism is less likely to develop into neural injuries in areas related to learning and memory.^132^ Concordantly, Park et al.^17^ reported that brain hypometabolism and neurodegeneration, assessed by FDG‐PET and MRI‐derived hippocampal volume and cortical thickness, are significantly associated with Aβ deposition as a proxy for AD progression in female but not in male older adults. In their longitudinal cohort, only women exhibit a consistent negative relationship between cerebral glucose metabolism and amyloid burden, a finding that is in line with FDG‐PET animal studies.^139^ Transcriptomic analyses of *post mortem* brains using 2 independent datasets further show that female AD patients have nearly nine‐fold more differentially expressed genes than males in the cortex, including 26 glucose‐metabolic genes (such as: *GOT1*, *GPI*, *PGAM1*, *ALDOA*) enriched in glycolysis/gluconeogenesis, adenosine monophosphate‐activated protein kinase (AMPK), and insulin signaling pathways, and the majority of them are downregulated. Among these, ENO2, PRKACB, and PPP2R5D are directly linked to APP and MAPT, indicating that dysregulated glucose metabolism in women may interact with amyloid and tau pathology. KEGG pathways related to glycolysis and gluconeogenesis are specifically regulated in women in this study.^17^ However, the functional Warburg‐like shift to glycolysis is shown to not be sex‐dependent in iPSC‐derived forebrain‐like neurons and astrocytes from human AD subjects in another study.^83^ Women exhibit a distinct neuro‐metabolic resilience pattern in early AD.^133^ Using data from 1259 male and female participants aged 55–90 years, five FDG‐PET defined regions of interest (bilateral posterior cingulate, bilateral angular gyri, and middle/inferior temporal gyrus) along with amyloid burden and hippocampal volume were analyzed. Findings suggest that women consistently have higher cerebral glucose metabolism than men when AD pathology is minimal to moderate, despite comparable amyloid and atrophy levels. This metabolic advantage coincides with superior cognitive performance across domains of global cognition, memory, and executive function which attenuates or disappears after adjusting for brain metabolism, indicating that higher glucose utilization might help with women's cognitive resilience in preclinical and prodromal AD. However, this female advantage diminishes with more advanced disease stage, indicating a steeper metabolic and cognitive decline trajectory as the disease progresses. Therefore, women's elevated metabolic activity may temporarily compensate for early AD pathology, but its eventual decline could contribute to their faster deterioration at later stages.^133^ Notably, whether such a fast deterioration could be a result of hormonal changes after menopause was not investigated. In agreement with these findings, a metabolomics and proteomics study on the *post mortem* hippocampus reveals that HIF‐1α signaling and TCA cycle are upregulated in healthy women versus healthy men, while the same pathways were downregulated in AD women versus AD men, suggesting a sharp reduction of the female advantage in these pathways during AD progression.^146^ Response to insulin and glucagon‐like‐peptide‐1 (GLP‐1) regulation of insulin secretion are other downregulated pathways in female compared to male AD *post mortem* hippocampi.^146^ Such an overturn of the female bioenergetic advantage can partly be due to hormonal changes, a concept which is discussed in the following sections of this paper. Furthermore, brain regions with glucose hypometabolism in AD can be sex‐dimorphic.^147^ Using voxel‐based FDG‐PET analysis, Nerattini et al.^147^ suggest that both sexes show classical AD‐related hypometabolism in parieto‐temporal and limbic areas, yet with distinct topographical distributions: women exhibited greater anterior (frontal and limbic) hypometabolism, whereas men show more posterior (parietal and precuneus) reductions. Supported by findings of region‐specific hypometabolism between men and women,^135^ the distinct hypometabolic patterns observed in AD can possibly reflect a sex‐specific vulnerability of brain regions that start with lower baseline metabolic activity, that is, anterior in females and posterior in males. This concept needs to be further explored and validated using longitudinal studies.

### 3.3 Sex‐specific brain mitochondrial function and efficiency in AD

Mitochondria play a pivotal role in metabolism and are identified as being sex‐dimorphic in AD.^83^, ^140^, ^141^, ^148^, ^149^, ^150^, ^151^ The 3xTg mouse model has been extensively studied in this context. In the brains of 18‐month‐old male 3xTg mice, cortical synaptic mitochondrial Complex IV is activated along with an increase expression level of Complexes II and III, indicating a possible hypermetabolic state in these neurons with high energy demand. In the females of the same age, however, cortical synaptic mitochondrial Complex I activity is inhibited while non‐synaptic Complex II activity is increased compared to wild‐type (WT) controls.^140^ These changes are accompanied by reduced expression of Electron Transport Chain (ETC) complexes I, III, and IV in females. Activation of non‐synaptic Complex II in females can possibly indicate heightened fatty acid metabolism in astrocytes, donating reducing equivalents to succinyl‐CoA and fueling succinate dehydrogenase (Complex II).^140^ Lipid peroxidation and ketone body utilization are also shown to be increased in the hippocampus of female 3xTg mice after a decline in glucose transport and metabolism, which can potentially augment oxidative stress.^51^, ^152^ Sex‐specific mitochondrial bioenergetic dysfunction is also reported in the hypothalamus of 3xTg mice.^141^ Hypothalamic mitochondrial ETC function is impaired as early as 2 months of age in the females, while such impairment does not occur until 6 months in the males.^141^ The expression levels of ETC complexes were also lower at 13‐month‐old females than males, suggesting the female vulnerability is persistent across age in this mouse model. In addition, female mice showed higher NF‐κB, pIκB‐α, and Nrf2 inflammatory biomarkers at different age groups during normal aging and AD progression.^141^ Mitochondrial bioenergetic deficits are suggested to happen before Aβ plaque buildup in the hippocampus of female 3xTg mice.^152^ Such a dysfunction is reflected in reduced pyruvate dehydrogenase expression, lowered OXPHOS activity, increased lipid peroxidation, and heightened free radical leak from the mitochondria as early as 3 months of age, when Aβ pathology is absent in the hippocampus.^152^

Building on the pre‐clinical investigations, a human iPSC‐based study provides evidence supporting the idea that mitochondrial dysfunction in AD is both cell type‐ and sex‐specific.^83^ The study uses iPSC lines derived from fibroblasts of male and female donors—either cognitively normal or diagnosed with sporadic AD—and differentiates them into neurons, astrocytes, and cerebral organoids. Both neuronal and astrocytic populations display a shift from OXPHOS to glycolysis regardless of sex, probably due to an uncoupling of the mitochondrial respiration that leads to suppressed ATP production. However, mitochondrial respiration assessed by Seahorse revealed that complex IV activity is significantly reduced in AD female, but not male, neurons and cerebral organoids. Neurons and astrocytes from females exhibit hyperpolarized mitochondrial membranes and elevated mitochondrial calcium levels, whereas those from males show depolarization without calcium accumulation.^83^ Elevated mitochondrial calcium can promote oxidative stress, protein misfolding, OXPHOS uncoupling, and apoptosis.^151^, ^153^ Mitochondrial localization near the lysosomes is also higher in female versus male AD astrocytes, indicating a greater need for the mitochondrial turnover and enhanced mitophagy.^83^ A deficit in Complex IV activity without a corresponding decline in complexes I and III is implicated to cause ROS leak and damage cellular compartments, which could be one of the reasons for mitochondrial membrane hyperpolarization in females.^154^ Beyond this, a recent single nucleous transcriptomics atlas of the human prefrontal cortex in AD shows several Complex I–related mitochondria‐encoded genes being upregulated in female and downregulated in male excitatory neurons.^148^ Such a mitochondrial response might be compensatory to counteract the overall energetic deficit observed in AD, which may eventually exacerbate ROS production. However, these findings should be interpreted with caution since single‐nucleus RNA‐seq measures nuclear‐retained RNA and captures only a variable subset of mitochondrial transcripts that adhere to nuclei. Also, since nuclear RNA retention itself varies across cell types, these mitochondrial expression differences could partially reflect RNA compartmentalization biases rather than true biological shifts. Functional measurement of the mitochondrial ETC activity may better elucidate such sex differences. A recent post‐mortem study of the prefrontal cortex in AD patients revealed a greater decline in mitochondrial complexes III and IV respiration in females compared to males.^149^ Males, in contrast, exhibited relatively preserved mitochondrial respiration but showed increased phosphorylation of mTOR at Ser2448, which can indicate insulin‐signaling dysregulation rather than respiratory failure. Furthermore, females show higher interleukin‐2 (IL‐2) levels and increased Iba‐1 immunoreactivity in white matter, pointing to exacerbation of inflammatory and microglial activation likely due to their greater mitochondrial dysfunction.^149^ Mitochondrial sex‐differences are also reported in the context of cerebrovascular impairment in the temporal cortex.^150^ Hypoxia, nutrient deprivation, and oxidative damage due to vascular dysfunction in AD can lead to proteome alterations such as increased citrullination of arginine and deamidation of glutamine residues of myelin basic protein (MBP) in a female‐specific pattern. These changes result in reduced MBP degradation and buildup of myelin components in white matter, which can disrupt axon‐glia metabolic coupling. Such alterations were accompanied with a reduction in ATP synthase subunits, cytochromes, as well as NADH‐ubiquinone proteins in females only.^150^

### 3.4 Interaction between glucose hypometabolism and Aβ/tau pathology in males versus females

Emerging multimodal imaging studies reveal that the relationships among Aβ deposition, tau pathology, and cerebral glucose metabolism differ markedly between sexes.^17^, ^18^, ^155^, ^156^, ^157^ Although some studies indicate similar brain Aβ load in women and men in AD,^158^, ^159^, ^160^ others report higher brain Aβ burden in women^161^, ^162^, ^163^, ^164^ including a recent study which used a more accurate quantitative threshold rather than a visual read to identify amyloid positivity.^164^ Additionally, cognitively normal women during peri‐menopause and post‐menopause stages are shown to have higher brain Aβ than men at the same age.^18^ Higher Aβ load—especially in the frontal cortex—is associated with glucose hypometabolism in women but not in men.^17^, ^18^ It has also been shown that plasma Aβ42/40 have positive correlations with brain FDG‐PET uptake only in women.^17^

Among amyloid‐positive individuals, women exhibit higher tau retention than men in the entorhinal, superior parietal, occipital, and inferior temporal cortices, as well as amygdala.^156^, ^157^, ^165^ Evidence shows that tau‐PET signal, not Aβ, best aligns with regional hypometabolism and they preferentially affect brain areas vital for cognitive functions including the entorhinal cortex.^166^, ^167^ From a mechanistic point of view, pathological tau impairs mitochondrial trafficking, reduces OXPHOS, and decreases ATP production in neurons.^168^ Reduced GLUT function at the BBB, as shown in women, is also shown to induce tau hyperphosphorylation in the frontal cortex.^72^ Therefore, it is conceivable that tau‐hypometabolism relationship might be stronger in women in areas related to cognitive task during AD progression. This needs further investigation in future longitudinal studies.

### 3.5 Role of sex hormone changes in brain glucose metabolism in AD

#### 3.5.1 Menopause and the effects of estrogen decline in females

The menopausal transition is marked by reproductive senescence and a decline in ovarian hormone production, particularly estrogen. Estrogen is well known to exert neuroprotective properties,^169^, ^170^, ^171^ especially in brain regions responsible for higher‐order cognitive functions such as the hippocampus,^172^, ^173^ and cerebral cortex.^174^, ^175^, ^176^ The neuroprotective role can be, in part, due to the vast regulatory functions of estrogen in brain glucose metabolism, including glucose uptake, glycolysis, and mitochondrial ATP production within the brain.^169^ Thus, the decline in this hormone at menopause likely contributes to the abnormalities in brain glucose metabolism.

Beyond chronological aging, endocrine aging emerges as a crucial determinant of brain metabolic decline. Mosconi et al.^18^ examined AD‐related endophenotypes—including increased Aβ deposition, cerebral glucose hypometabolism, and reductions in gray and white matter—across successive endocrine transition stages in cognitively normal women, and compared these patterns both across stages and to those observed in men. They targeted posterior cingulate cortex, precuneus, inferior and superior parietal lobule, lateral and medial temporal cortex, and medial, inferior, and prefrontal cortex. In the peri‐menopausal stage, women generally exhibited lower glucose metabolism in the frontal cortex compared to men. This reduction was even lower during the post‐menopause stage. In both peri‐ and post‐menopausal women, glucose metabolism is reduced in the posterior cingulate/precuneus, and parietal and temporal cortex compared to women at reproductive age or to men. The findings are robust even after adjusting for education and *APOE* status. With the same pattern, Aβ deposition was higher and the white matter volume was lower in peri‐ and post‐menopausal women compared to men and pre‐menopausal women.^18^ However, such results need to be confirmed by longitudinal studies with a larger sample size and quantitative measurement of estrogen. Animal studies have reported findings consistent with the human data^177^, ^178^; irregular estrus cycling in rodents is endocrinologically similar to peri‐menopause stage with reduced cycle frequency and diminished fecundity.^179^ Rats with irregular estrus cycle are reported to have reduced brain glucose metabolism as shown by reduced whole brain FDG uptake, respiratory control ratio, ATP synthase, and cyclooxygenase (COX) activity.^177^ These changes are accompanied by downregulated expression of hippocampal genes involved in glucose transport and glycolysis, as well as an increase in brain H_2_O_2_ levels indicating higher oxidative damage.^177^ Ovariectomy, a less physiological type of ovarian hormone depletion than menopause, is shown to result in memory and learning impairment in C57BL/6J mice after 3 months. Of note, mitochondrial dysfunction—including a drop in complexes I, II, and IV—emerges several weeks before the onset of cognitive deficits, suggesting that impaired mitochondrial function may underlie the memory decline observed following ovariectomy.^178^

Based on findings from a metabolomics and proteomics study, estrogen receptor β signaling, along with TCA cycle and HIF‐1α signaling, were higher in the hippocampus of healthy female individuals compared to their male counterparts but were significantly lower in female AD than those in male AD patients.^146^ Such a pattern suggests a potential link between the decline in estrogen signaling and brain glucose metabolism in AD. This notion is consistent with the findings of reduced brain bioenergetics due to suppressed estrogen levels in 3xTg mice.^180^, ^181^ In this model, estrogen depletion induced by ovariectomy leads to a marked reduction in mitochondrial respiratory capacity, heightened oxidative stress, and accumulation of mitochondrial Aβ and Aβ‐binding alcohol dehydrogenase at 6 months of age.^180^ It also promotes a shift in mitochondrial dynamics favoring fission over fusion. Notably, these effects are more pronounced in 3xTgAD mice than in non‐transgenic controls, indicating that estrogen loss exacerbates existing AD‐related mitochondrial vulnerability. Such deficits were prevented by 3 months of estrogen replacement just before sacrifice.^180^ Another bioenergetic change seen in ovariectomized 3xTg mice is a shift away from utilization of glucose towards alternative substrates.^181^ In line with this, ovariectomy results in a significant reduction in brain glucose uptake, a downregulation of BBB glucose transporters, and a decline in hexokinase expression and activity. This reduction in glucose availability is coupled with increased glial lactate generation and use, elevated circulating ketone bodies, and upregulation of neuronal monocarboxylate transporter‐2 and 3‐oxoacid‐CoA transferase for ketone disposal and utilization. Importantly, these metabolic alterations occurred alongside elevated Aβ oligomer accumulation. Estrogen replacement in this model preserves glucose‐driven metabolic capacity, attenuated the shift toward alternative fuel reliance, and partially reduced Aβ oligomer burden.^181^ Evidence from a longitudinal study in female 3xTgAD mice further supported the concept of an early bioenergetic transition in the aging female brain even without ovariectomy.^51^ Simultaneous with the reproductive senescence transition, both WT and 3xTg females exhibit a decline in brain glucose transport on FDG‐micro‐PET beginning around 6–9 months of age, accompanied by reduced neuronal glucose transporter expression, decreased hexokinase activity, and increased phosphorylation (inactivation) of pyruvate dehydrogenase. In contrast to the ovariectomized model, where lactate was utilized more as an alternative fuel, its metabolism in this ovary‐intact AD mice diminished in parallel with a reduction in glucose transport.^51^ Since lactate import into neurons is essential for forming LTP and long‐term memory,^29^ such a shift in AD models of estrogen depletion might contribute to the cognitive decline. In both 3xTg and WT females, this deficit is met by an adaptive increase in ketone body transport and utilization. However, the shift happens earlier in life and subsequently deteriorates with age in 3xTg mice. Also, monocarboxylate transporter expression increases in neurons and decreases at the BBB and in astrocytes, effectively prioritizing ketone delivery to neurons at the expense of system‐wide metabolic support.^51^ Whether a similar shift toward ketone body metabolism occurs in humans with sporadic AD and what fatty acid sources the brain uses to meet their energy requirement needs further investigation.

Menopause‐associated estrogen withdrawal diminishes energy support when AD‑related hypometabolism occurs.^182^ The sharp fall in estrogen temporally aligns with the midlife emergence of AD endophenotypes in women, including reduced brain glucose metabolism and activation of amyloidogenic and inflammatory processes that precede clinical AD. Therefore, hormone replacement therapy has been suggested as a viable option to prevent AD in women when initiated early in relation to menopausal transition.^18^, ^182^, ^183^, ^184^ Neuroimaging studies in cognitively intact women reveal that those currently receiving estrogen replacement therapy have the highest rates of cerebral glucose metabolism, with values significantly greater than those observed in women with AD.^185^ By contrast, women who have never used estrogen therapy show metabolic rates that are intermediate but statistically indistinguishable from women with AD, suggesting that estrogen depletion is associated with an AD‐like metabolic profile.^185^ Consistent findings have emerged from a longitudinal study of healthy post‐menopausal study participants in which women not receiving estrogen therapy experienced a significant decline in glucose metabolism within the posterior cingulate cortex, while women on hormone therapy exhibited no such decline.^186^ On the other hand, studies show an increased risk of developing AD in women who initiated hormone replacement therapy after a long period of estrogen deprivation.^187^, ^188^ Recent tau‐PET data complement these findings by demonstrating that cognitively intact, post‐menopausal women show higher tau deposition than age‐matched men at equivalent Aβ levels, particularly when menopause occurs earlier or hormone therapy is initiated late.^189^ These observations suggest that prolonged estrogen deprivation may exacerbate tau vulnerability in the presence of amyloid pathology. Thus, there seems to be a “critical window” for initiating hormone therapy before or around the time of menopause when the central nervous system (CNS) function is largely intact, but hormone therapy may be detrimental when started years later when neurobiological decline has already begun.^190^, ^191^

#### 3.5.2 Effects of testosterone decline in males

Unlike the abrupt hormonal shift during menopause, age‐related testosterone decline in men is gradual, heterogeneous, and not organized around a discrete neuroendocrine transition. It may interact with metabolic aging and neurodegenerative vulnerability in men.^192^, ^193^ Across experimental and translational models, testosterone deficiency has been linked to changes in mitochondrial function, redox balance, and metabolic regulation in the brain.^194^, ^195^, ^196^, ^197^ Accordingly, findings from a human aging cohort suggest that testosterone may support trajectories of brain glucose metabolism and cognitive vulnerability later in life.^198^ In a longitudinal cohort of non‐demented older male and female adults, higher baseline testosterone in men—but not women—was associated with a slower rate of decline in global and regional brain glucose metabolism over time.^198^ Testosterone is also linked with lower AD incidence.^199^, ^200^ A systematic review and meta‐analysis of prospective cohort studies reported that low plasma testosterone levels were associated with a significantly increased risk of AD (pooled RR ≈ 1.5).^199^ Also, a more recent large UK Biobank study demonstrated that lower total testosterone predicted a higher incidence of all‐cause dementia and AD in a graded fashion.^200^ However, no studies to date have tested whether testosterone's protective effect against AD is mediated through its role in glucose metabolism. Mechanistic and experimental studies provide biological support for these associations. In rodents, gonadectomy leads to marked reductions in cytochrome‐c oxidase subunit 3 protein levels and multiple mitochondrial DNA‐encoded respiratory chain transcripts in the hippocampus^197^ and nigrostriatal dopaminergic system,^196^ whereas testosterone replacement restores mitochondrial gene expression, enhances antioxidant enzyme activity, and improves mitochondrial function.^195^, ^196^, ^197^ In AD‐relevant models, testosterone deficiency induced by castration worsens spatial learning and memory deficits and increases hippocampal histopathology in male APP/PS1 mice, accompanied by higher oxidative damage, reduced mitochondrial membrane potential, lower Complex IV activity, impaired mitochondrial biogenesis and dynamics, and accumulation of defective mitochondria.^201^ Testosterone pre‐treatment in neuronal cultures attenuates oxidative stress‐induced mitochondrial dysfunction through an androgen receptor‐dependent mechanism, which underlines a direct link between androgen signaling and mitochondrial homeostasis under a pathological stress.^201^ Parallel lines of evidence indicate that androgen depletion increases Aβ burden and impairs cognition, whereas testosterone or dihydrotestosterone therapy can reduce Aβ levels and improve behavioral performance in experimental models.^202^ In 3xTg male mice, aging is associated with progressive Aβ accumulation, and gonadectomy accelerates Aβ deposition and aggravates hippocampal‐dependent cognitive impairment. Of note, treatment with the non‐aromatizable androgen dihydrotestosterone attenuates both Aβ pathology and behavioral deficits, implicating androgen receptor‐specific mechanisms independent of its conversion to estrogen.^203^ Beyond amyloid‐ and mitochondria‐centric mechanisms, androgen signaling also intersects with metabolic and insulin signaling pathways that are tightly coupled to brain bioenergetics. Evidence from a neuronal androgen receptor knockout model demonstrates that the hypothalamic androgen receptor plays a critical role in insulin signaling. Deletion of androgen receptors in the hypothalamus can induce hypothalamic insulin resistance and exacerbate systemic metabolic syndrome, which leads to potential lowering of metabolic resilience in the aging male brain.^193^ Finally, proteomics work in middle‐aged C57BL/6J mice shows that gonadectomy alters hippocampal mitochondrial protein networks in a sex‐dependent pattern: in males, hormone loss produces larger disruptions in OXPHOS/respirasome complexes (particularly Complex I, IV, and ATP‐synthase), whereas in females, the predominant effects occur in mitochondrial translation, ribosomal, and chaperone pathways rather than core OXPHOS structure.^194^ Tibolone (a synthetic steroid medication) only partially reverses these effects, indicating that male and female mitochondrial networks rely on distinct hormone‐linked regulatory architectures.^194^ These findings support a model in which androgen decline in men does not trigger a single metabolic transition state but may instead erode mitochondrial and metabolic buffering capacity over time, thereby amplifying vulnerability to AD‐related pathology when other biological stressors are present. The limited number of mechanistic findings in men compared to the menopause literature in women may be attributed in part to historical patterns of clinical practice: testosterone replacement therapy has been used far less commonly in men than menopausal estrogen therapy in women, limiting opportunities for large‐scale, treatment‐informed observational and interventional studies.^192^ This asymmetry underscores an important gap in the field and highlights the need for sex‐balanced endocrine–brain metabolic research to clarify whether preserving androgen signaling in aging men can meaningfully support bioenergetic resilience and modify trajectories of AD risk.

### 3.6 Role of genetic factors in sex‐specific glucose metabolic alterations in AD

#### 3.6.1 Sex‐dependent metabolic effects of *APOE* gene

The *APOE* gene is the best‐known sporadic genetic risk factor for AD. It is a polymorphic gene located on chromosome 19, which can exist in three variants: *ε2*, *ε3*, and *ε4* distinguished by variations at amino acid positions 112 and 158.^204^ Of these, the *APOE ε4* allele confers a major genetic risk for late‐onset AD, compared to the more common *APOE ε3* isoform,^205^ while the *ε2* variant offers some protective effect, making it the least associated with AD risk.^206^, ^207^ Compared to individuals carrying *APOE ε3/ε3* genotype, AD risk is increased threefold in *APOE ε3/ε4* carriers, and 15‐fold in *APOE ε4/ε4* carriers.^208^ The exact mechanisms by which different *APOE* alleles influence Aβ and tau pathologies, as well as neuroinflammation and neurodegeneration, are still under intensive investigation and remain a critical focus of current research.^209^ Although the protective *APOE ε2* genotype is reported to have mixed sex‐dependent effects,^1^, ^210^ *APOE ε4* isoform is associated with more aggravated disease progression in women compared to men.^211^, ^212^ The elevated AD risk associated with *APOE* ε*4* might be driven primarily by its stronger impact in women, since a meta‐analysis reported that women carrying a single *APOE ε4* allele had a four‐fold higher risk of developing AD compared to women with two *APOE ε3* alleles. In contrast, men with one *APOE ε4* allele showed little to no increase in risk.^155^ However, another meta‐analysis found no difference between sexes in the overall relationship between *APOE* genotype and AD.^213^ Intriguingly, the analysis noted that age could be pivotal, with *APOE ε4* linked to higher AD risk in women compared to men between the ages 55 and 70, when *APOE* is believed to exert its greatest influence.^213^ Besides age, the female vulnerability to *APOE ε4* might depend on the disease stage. *APOE ε4* may heighten female‐biased tau deposit in MCI patients in that female *ε4* carriers show higher tau levels in the cerebrospinal fluid and higher tau/Aβ ratio than male *ε4* carriers at similar Aβ levels.^155^ However, tau accumulation is not significantly different between cognitively healthy male and female *APOE ε4* carriers.^156^ These data suggest the female vulnerability to *APOE ε4* impact might be linked with the progression of tau pathology.

*APOE ε4* allele is known to disturb brain glucose metabolism. In a PET study of cognitively normal adults aged 47–68 years, including *ε4* homozygotes, heterozygotes, and noncarriers, having more copies of the *ε4* allele was shown to be associated with lower glucose metabolism in the precuneus and the posterior cingulate, parietotemporal, and frontal cortex.^214^ Perkins et al.^215^ reported that young adult carriers of the *ɛ4* allele show early alterations in energy metabolism pathways in the posterior cingulate region, aligning with changes in glucose, ketone, and mitochondrial energy metabolism pathways, which suggests metabolic dysfunction is present well before clinical symptoms of AD arise in *APOE ɛ4* carriers.^215^ *APOE ɛ4* has also been reported to shift brain metabolism from OXPHOS to aerobic glycolysis in mice expressing human *APOE ɛ4*.^216^ Consistent with these observations, *APOE ɛ4* hiPSC‐induced astrocytes and hiPSC‐induced neurons show AD‐like glucose metabolic signature including heightened glycolytic flux, increased labeling of glycolytic and nucleotide intermediates, and elevated O‐GlcNAcylation regardless of sex. These cells also demonstrate mitochondrial dysfunction, including impaired TCA cycle metabolism and dysregulated OXPHOS.^217^

Sex and *APOE* genotype are reported to interact for the control of brain glucose metabolism.^216^, ^218^, ^219^, ^220^, ^221^, ^222^, ^223^ *APOE ε4* allele amplifies sex‐specific differences in glucose metabolic gene regulation. Transcriptomic analyses of the hippocampal genes in human‐*APOE ε4‐*transgenic (*hAPOE ε4*) mice revealed a metabolic signature characterized by increased fatty acid beta oxidation and impaired mitochondrial gene expression in *APOE ε4* female mice.^221^ In contrast, male *APOE ε4* mice exhibited a profile skewed toward enhanced glycolysis and TCA cycle activity, reflected in the upregulation of genes involved in oxidative metabolism.^221^ A shift to aerobic glycolysis and away from TCA cycle and OXPHOS in *hAPOE ε4* female mice has been replicated in another study which shows these effects particularly in the astrocytes by transcriptomics, metabolomics, and glucose‐labeling techniques. Further, a reduction in the whole‐body energy expenditure and oxygen consumption rate along with elevated plasma lactate levels seen in cognitively healthy young female *APOE ε4* carriers but not male carriers suggest that such a metabolic shift might occur across the whole organism before pathology sets in.^216^ Consistent with this reprogramming, *ε4*‐positive women exhibit a buildup of medium‐chain acylcarnitines in association with higher phosphorylated tau, suggesting that although lipid‐derived fuel pathways are recruited, mitochondrial β‐oxidation becomes inefficient under increased energy demand.^220^ These findings support a model in which female *APOE ε4* carriers engage compensatory shifts toward glycolytic and alternative fuel metabolism, but ultimately experience incomplete or energetically fragile mitochondrial adaptation, potentially raising the vulnerability to downstream AD pathology. Importantly, emerging evidence suggests that the metabolic consequences of *APOE ε4* are strongly modulated by the midlife endocrine transition in females, with peri‐menopause acting as a critical window of bioenergetic vulnerability (see Section 3.5). In a recent animal study using a peri‐menopause model framework, *APOE ε4* females failed to mount the adaptive metabolic reprogramming observed in *APOE ε3* counterparts. Instead, they display a progressive mitochondrial dysfunction, impaired AMPK‐PGC‐1α signaling, increased neuroinflammation, and demyelination.^219^ These findings support the view that menopause‐linked endocrine decline unmasks or amplifies *APOE ε4*‐related metabolic fragility in females.

*APOE ε4* is negatively associated with brain glucose metabolism measured by FDG uptake in cognitively normal men but not women with discernable memory issues.^222^, ^223^, ^224^ Nevertheless, while cognitively normal female *APOE ε4* carriers with no memory problems have modest reductions in brain glucose metabolism compared to non‐carriers,^223^, ^224^ females with significant memory concern (non‐MCI and non‐demented) have shown an elevation in brain glucose metabolism relative to their non‐carriers in temporal and parietal regions, This reported observation could be a female‐specific attempt to maintain normal brain function despite a more severe pathology.^222^ Complementing this, a ^31^P‐magnetic resonance spectroscopy (MRS) study in cognitively normal midlife adults at risk for AD reported that women already exhibited higher ATP utilization and energy demand (lower PCr/ATP and PCr/Pi) in AD‐vulnerable cortices relative to men, with *APOE ε4* further accentuating these bioenergetic alterations.^218^ Therefore, the elevated FDG‐PET signal observed in *ε4*‐positive women may represent a compensatory upregulation of glucose metabolism superimposed on an underlying mitochondrial energy deficit, potentially helping to maintain cognitive performance during early disease stages. Sex‐specific effect of *APOE ε4* on brain glucose metabolism seems to dilute with disease progression.^223^ A cross‐sectional study found *APOE ε4* to be inversely associated with brain glucose metabolism in MCI regardless of sex, whereas such an association was not evident in AD. This observation might suggest that the pathology at advanced stages outweigh *APOE ε4*‐related effects on brain metabolism.^223^

#### 3.6.2 X‐Linked genetic modulators of brain glucose metabolism in AD

The X chromosome can be an important point of distinction in AD pathology, since it is enriched in genes necessary for neural development^225^, ^226^ and is associated with slower cognitive aging in the female brain.^226^ Whether X‐linked genes can affect brain glucose metabolism in a sex‐dependent way is an interesting and critical, yet less‐studied domain that calls for further investigation. Although both males (XY) and females (XX) possess a single active X chromosome as a result of X‐chromosome inactivation in females,^227^ a subset of X‐linked genes escape transcriptional X‐silencing, leading to relatively lower mRNA expression in males than females.^228^, ^229^, ^230^ One such gene with a role in modulating metabolism is *KDM6A*.^231^, ^232^, ^233^ The canonical role of this gene is through demethylation of the transcription factor histone H3 lysine 27 (H3K27), thus activating the expression of target genes.^231^, ^232^ *KDM6A* has been shown to transcriptionally induce aerobic glycolysis in brain glioblastoma, thereby increasing tumor growth.^231^ This canonical pathway has also been shown to suppress nerve and axonal regeneration both in peripheral and central nervous systems.^234^ Thus, one might expect *KDM6A* overexpression to be detrimental for the brain function in AD. However, *KDM6A* overexpression is shown to confer cognitive resilience in both male and female transgenic mice overexpressing human *APP* gene and in humans carrying a minor allele of the gene.^235^ In addition, overexpression of this gene reduced Aβ toxicity while its deletion increased toxicity in neurons in vitro, further confirming its neuroprotective role.^235^ Interestingly, this protective role is suggested not to be through the canonical role of this gene as a demethylase, since an engineered form of *KDM6A* lacking demethylase function is also reported to enhance cognition.^225^ The X‐chromosome escapee genes increase with age, suggesting that epigenetic control of the inactive X may relax over the lifespan.^230^ *KDM6A* is shown to escape and thus be more expressed in women than men.^235^ It is also upregulated in AD compared to cognitively normal individuals of both sexes in the temporal and parahippocampal cortex.^235^ We may speculate that the escape of this gene, especially its minor allele, might be the mechanism behind women's cognitive resilience before AD pathology begins. However, it might be to their detriment in a chronic way due to the simultaneous canonical upregulation through enhancing a glycolytic shift over OXPHOS, hampering with neural regeneration, and diminishing cognitive resilience in AD. This represents a promising hypothesis that warrants investigation in future studies.

**FIGURE 2:**
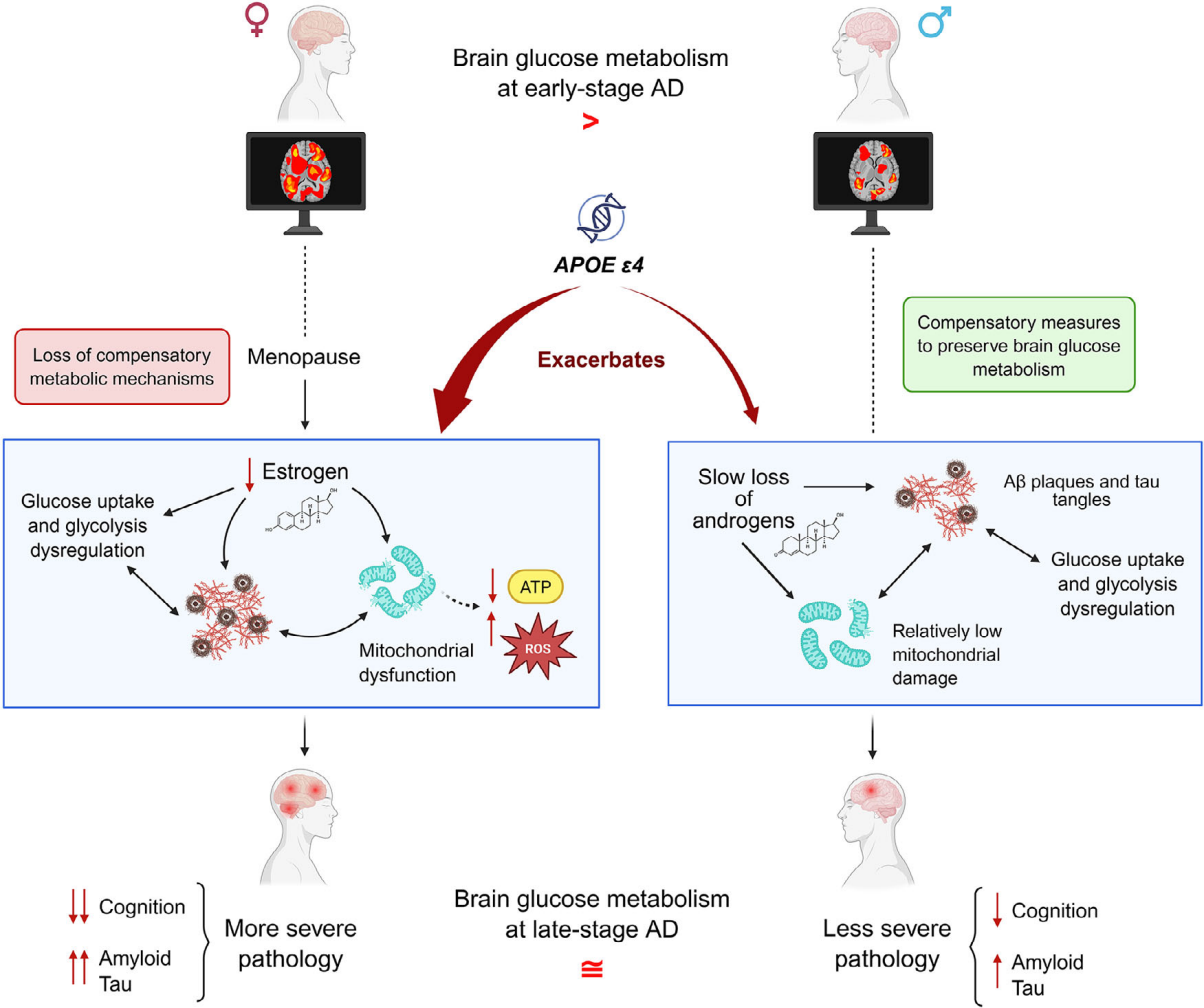
Sex differences in brain glucose metabolism in AD. Sex differences in brain glucose metabolism in AD. Women have a metabolic advantage at early stages of AD; however, amyloid and tau, abrupt hormonal changes in menopause, glucose uptake and glycolysis dysregulation, and failure of compensatory measures lead to mitochondrial damage, which can exacerbate amyloid and tau pathology in a feed‐forward loop. Mitochondrial dysfunction reduces ATP production and heightens oxidative stress, eventually leading to faster progression of hypometabolism and more severe pathology. Amyloid and tau are milder, and the loss of sex hormones is slower in men. They experience relatively less mitochondrial damage, leading to better metabolic compensation and maintaining brain glucose metabolism, resulting in slower disease progression and less severe pathology. *APOE ε4* exacerbates glucose metabolic abnormalities in both sexes, more harshly in women. ↑, increase; ↓, reduction; →, leads to; AD, Alzheimer's disease; *APOE*, apolipoprotein E; ATP, adenosine triphosphate.

Figure 2 depicts an illustration of sex differences in brain glucose metabolism happening in AD.

## 4 CONCLUSION

A consistent picture emerges across molecular, imaging, and animal studies in AD field: brain glucose hypometabolism reflects a failure of resilience rather than a single pathway defect, with evidence that this failure unfolds differently in women and men. Integrated evidence across human, animal, and cell models suggests that impaired glucose transport, disrupted glycolysis, mitochondrial dysfunction, and brain insulin resistance underlie hypometabolism in AD. These alterations likely act in a bidirectional feed‐forward loop, both driven by and accelerating Aβ and tau pathology, with indications that the decline in mitochondrial glucose metabolism is steeper and more pathologically aligned in females. Such female vulnerability is mainly attributed to the rapid estrogen decline in menopause as well as a higher susceptibility to *APOE ε4* genotype. A Warburg‐like shift toward glycolysis may serve as a transient compensatory mechanism in both sexes, yet its expression appears cell type‐ and sex‐dependent and may ultimately exacerbate metabolic stress, underscoring the need to determine when the compensation becomes maladaptation.

Most experimental studies do not yet resolve sex differences at the level of cell type, brain region, or disease stage, nor do they adequately disentangle early metabolic changes from those secondary to Aβ and tau. Likewise, numerous omics‐based discoveries await validation in functional in vivo and in vitro assays and translation into clinically meaningful biomarkers. Looking forward, several priorities are clear. First, sex‐stratified longitudinal human studies are needed to map when and where hypometabolism first emerges in the brain, and how trajectories differ across life stage, hormonal status, and *APOE ε4* genotype. Such work will be essential to identify optimal windows for prevention or intervention and to enable region‐specific and mechanism‐targeted strategies. Second, putative metabolic signatures, including peri‐ and post‐menopausal glycolytic shifts, require confirmation in humans with careful consideration of sex hormone levels. Third, endocrine context must be incorporated into study design: while menopause has been extensively interrogated, there remains a comparative scarcity of large‐scale observational and interventional research on androgens, brain metabolism, and cognition in men. And lastly, the X‐chromosome remains an underexplored regulator of metabolic vulnerability, and its interactions with sex hormones, *APOE*, and cell type‐specific metabolism warrant systematic investigation. These directions point toward a reframing of AD pathogenesis in which sex, endocrine aging, genetic risk, and cellular metabolism are treated as interacting axes rather than parallel domains. The progress will depend on integrating multi‐modal imaging with cell‐resolved mechanistic studies, linking metabolic phenotypes to functional outcomes, and developing testable, sex‐informed hypotheses around mitochondrial repair, cellular metabolic flexibility, and early stress buffering, particularly in women at elevated genetic and hormonal risk. Clarifying these pathways will be essential for advancing toward metabolic‐precision interventions capable of altering disease trajectories before the onset of the irreversible neurodegeneration.

## CONFLICT OF INTEREST STATEMENT

The authors have nothing to disclose. Author disclosures are available in the Supporting Information.

## Supporting information




**Supporting Information**: alz71491‐sup‐0001‐SuppMat.pdf
